# Germination and seed persistence of *Amaranthus retroflexus* and *Amaranthus viridis*: Two emerging weeds in Australian cotton and other summer crops

**DOI:** 10.1371/journal.pone.0263798

**Published:** 2022-02-09

**Authors:** Asad M. Khan, Ahmadreza Mobli, Jeff A. Werth, Bhagirath S. Chauhan

**Affiliations:** 1 Queensland Alliance for Agriculture and Food Innovation (QAAFI), The University of Queensland, Gatton, Queensland, Australia; 2 Department of Agrotechnology, Faculty of Agriculture, Ferdowsi University of Mashhad, Mashhad, Iran; 3 Leslie Research Centre, Queensland Department of Agriculture and Fisheries, Toowoomba, Australia; 4 School of Agriculture and Food Sciences (SAFS), The University of Queensland, Gatton, Queensland, Australia; 5 Chaudhary Charan Singh Haryana Agricultural University (CCSHAU), Hisar Haryana, India; Anhui Agricultural University, CHINA

## Abstract

Redroot pigweed (*Amaranthus retroflexus* L.) and slender amaranth (*Amaranthus viridis* L.) are becoming problematic weeds in summer crops, including cotton in Australia. A series of laboratory and field experiments were performed to examine the germination ecology, and seed persistence of two populations of *A*. *retroflexus* and *A*. *viridis* collected from the Goondiwindi and Gatton regions of Australia. Both populations of *A*. *retroflexus* and *A*. *viridis* behaved similarly to different environmental conditions. Initial dormancy was observed in fresh seeds of both species; however, germination reached maximum after an after-ripening period of two months at room temperature. Light was not a mandatory prerequisite for germination of both species as they could germinate under complete darkness. Although both species showed very low germination at the alternating day/night temperature of 15/5 C, these species germinated more than 40% between ranges of 25/15 C to 35/25 C. Maximum germination of *A*. *retroflexus* (93%) and *A*. *viridis* (86%) was observed at 35/25 C and 30/20, respectively. Germination of *A*. *retroflexus* and *A*. *viridis* was completely inhibited at osmotic potentials of -1.0 and -0.6 MPa, respectively. No germination was observed in both species at the sodium chloride concentration of 200 mM. *A*. *retroflexus* seedling emergence (87%) was maximum from the seeds buried at 1 cm while the maximum germination of *A*. *viridis* (72%) was observed at the soil surface. No seedling emergence was observed from a burial depth of 8 cm for both species. In both species, seed persistence increased with increasing burial depth. At 24 months after seed placement, seed depletion ranged from 75% (10 cm depth) to 94% (soil surface) for *A*. *retroflexus*, and ranged from 79% to 94% for *A*. *viridis*, respectively. Information gained from this study will contribute to an integrated control programs for *A*. *retroflexu*s and *A*. *viridis*.

## Introduction

Redroot pigweed (*Amaranthus retroflexus* L.) and slender amaranth (*Amaranthus viridis* L.) are becoming problem weeds of cotton (*Gossypium hirsutum* L.) cropping systems and other summer crops in Australia [1–3]. *A*. *retroflexus* is native to central and eastern USA and nearby regions of Canada, but it has naturalized to the temperate regions of Asia, Africa, Europe, and Australia [4]. It was classified as highly invasive in row crops, such as cotton, soybean [*Glycine max* (L.) Merr.], corn (*Zea mays* L.), sugar beet (*Beta vulgaris* L.), sorghum [*Sorghum bicolor* (L.) Moench], and several vegetable crops [5, 6]. As an example, the presence of 8 plants m-1 row of this weed could reduce snap bean (*Phaseolus vulgaris* L.) yield by up to 58% [7]. A computer search reveleved that no information exists in Australia on the effect of these weeds on crop yield. *A*. *retroflexus* is a prolific seed producer; a single plant of *A*. *retroflexus* can produce up to 300,000 seeds [8, 9].

*Amaranthus viridis* is an extensively prevalent weed in plantation agriculture. It is a weed in 50 crops and widely distributed in 80 countries of the world [4]. It is native to the USA and a very common weed of tropics, subtropics, and warm temperate regions all over the world [4]. *A*. *viridis* is also widely distributed in Australia, and is considered a major problem in New South Wales, South Australia, and Tasmania [1–3]. The presence of 12 plants m^2^ of this weed reduced the leaf area, leaf biomass, and stem biomass of red pepper (*Capsicum baccatum* L.) by 25%, 72%, and 74%, respectively [10]. A single plant of *A*. *viridis* can produce 7,000 seeds and the seedbank of *A*. *viridis* builds-up very fast in the absence of control [6].

Prolific growth and wide distribution of these weed species are favoured by their germination potential and high seed production ability in varied environmental conditions [5]. Besides having a C_4_ photosynthetic pathway and a high competitive potential [11], allelopathic compounds from these species could further enhance their invasiveness [12, 13]. Although there are no reports on herbicide-resistant biotypes of these weeds in Australia, the potential resistance to acetolactate synthase and photosystem II inhibitor herbicides, likewise the other parts of the world, could bring new challenges for the Australian crop production [14].

High seed production of *Amaranthus* species may excessively enrich the soil seedbank, which ensures their regeneration despite biotic and abiotic constraints and contributes to further infestations over time and locations [15, 16]. Weed seedbanks get depleted through predation, decay, and germination [17]. Although studies on seedbank persistence of *Amaranthus* species showed that less than 5% of the seeds were viable for more than 4 year, it seems that their ubiquitous presence contributes to their existence in many cropping systems worldwide [16, 18, 19]. Weed seeds in a persistent seedbank could remain viable for a long time in unpredictable conditions and emerge in several flushes during favorable conditions [17]. Therefore, studying seedbank persistence and germination behavior could be very important for the development of efficient weed management strategies.

Seed germination biology plays a significant role in the emergence and establishment of invasive weeds in an agroecosystem by impacting the availability of viable seeds in seedbanks [5, 20]. Environmental factors, such as temperature, light, moisture availability, and salinity influence the ability of weed seeds to germinate, survive, and establish in certain agroecosystems [20, 21]. Breaking dormancy and germination rates of many weed species are highly associated with temperature fluctuations [22]. It has been reported that a light requirement also may also inhibit germination in soil depth and under crop canopies in some species [20, 22, 23]. Water deficiency and soil salinity are major problems throughout Australia [24]; therefore, the evaluation of germination responses of weed species to these key environmental conditions is crucial. Weed seedling emergence depends largely on seed burial depth [20, 23]. Thereby, weed emergence can be suppressed by increasing the burial depth of seeds through deep tillage [20, 23]. A better understanding of environmental factors influencing the germination of weed seeds is necessary to devise a better control strategy.

Germination behavior of weed seeds in a wide range of environmental conditions determines the level of invasionand the demographics within a cropping system [20, 22]. Although *A*. *retroflexus* and *A*. *viridis* are members of the same family, their germination may respond differently to environmental conditions. A comparison of the germination behavior of weed species in various environmental conditions highlights their superiority of potential invasion and distribution in new areas [5]. Therefore, in the current study, germination responses of these species were compared to better understand the success of weed management practices. Although some information is available on the seedbank persistence and germination behavior of *A*. *retroflexus* and *A*. *viridis* in Asia and the USA [15, 16, 18, 21, 25, 26], information on Australian populations is not available, and comprehensive studies are needed before these weeds become more problematic. The objective of this study was to investigate the seedbank persistence and germination behavior of Australian populations of *A*. *retroflexus* and *A*. *viridis* in response to different temperatures, osmotic potentials, salt stress, and burial depths conditions.

## Material and methods

### Seed collection

**Fig 1:**
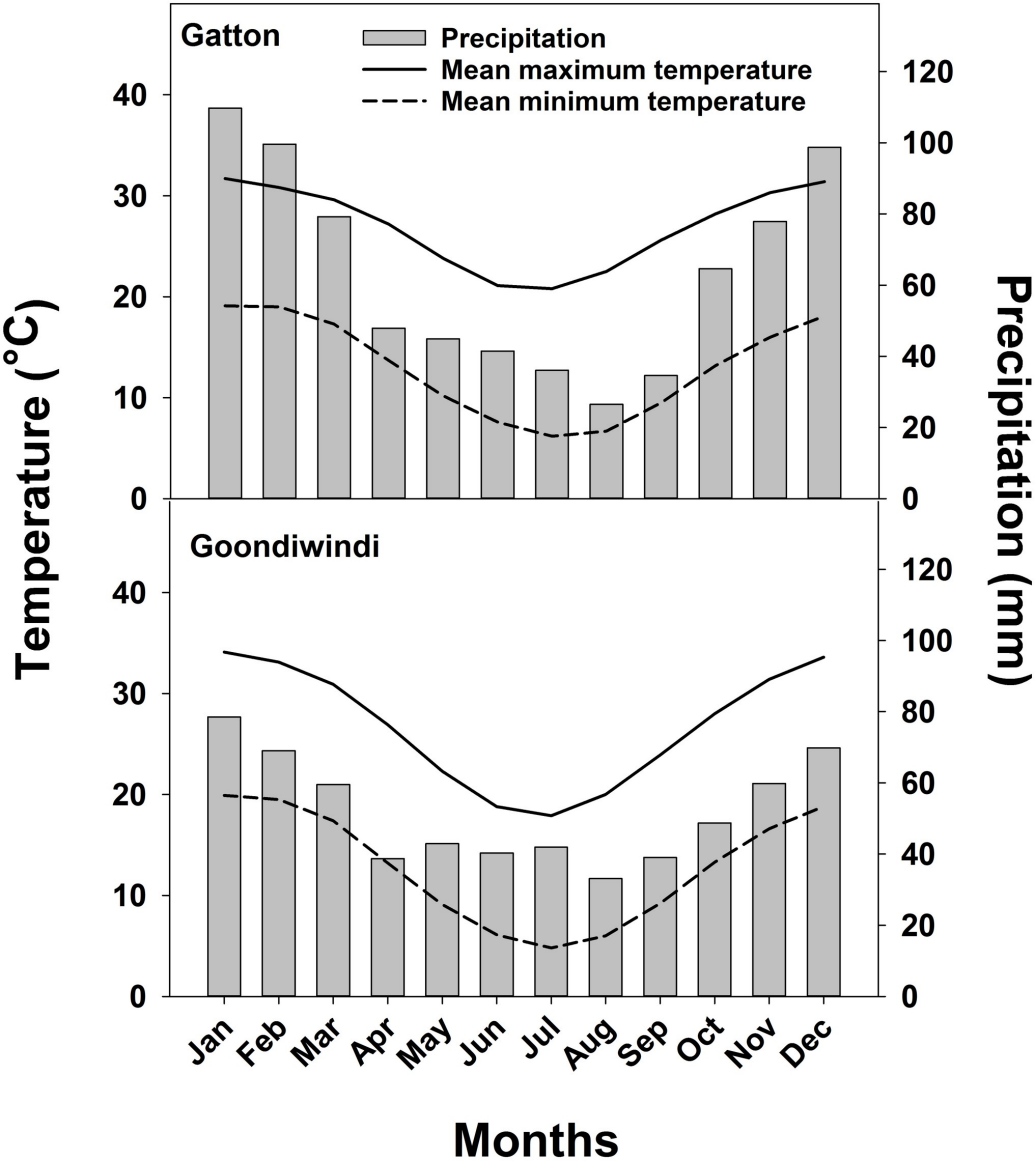
Long-term (100 years; 1918–2019) monthly mean precipitation and maximum and minimum temperatures for Gatton and Goondiwindi, Queensland, Australia.

Mature seeds of *A*. *retroflexus* and *A*. *viridis* were collected from Queensland’s regions of Goondiwindi (28.41° S, 150. 23° E) and Gatton (27.45°S, 152.21°E) in March 2017. Goondiwindi is 256 km (aerial distance) from Gatton, and its altitude is 217 m, whereas the altitude of Gatton is 94 m. Long-term (1918–2019) mean precipitation and maximum and minimum temperature data for both locations are shown in Fig 1. Gatton receives 765 mm of average annual rainfall whereas Goondiwindi receives an average annual rainfall of 620 mm. Fully mature seeds were collected from cropping fields (cotton and sorghun) by tapping the inflorescence gently in a tray. Each population was collected from 40–50 plants dispersed in a field of 3–4 ha. The seeds were dried, cleaned, and stored in paper bags. In September 2017, these seeds were planted at Gatton in the same environmental conditions to remove the effect of maternal environmental conditions [27] and fresh seeds were harvested in January 2018. The seeds of both species were stored at room temperature (25°C) in separate paper bags until the start of the experiments.

## Germination protocol

All the studies were conducted in the weed science facilities of the Queensland Alliance for Agriculture and Food Innovation (QAAFI), University of Queensland, Gatton, Australia. Twenty-five seeds of both populations of *A*. *retroflexus* and *A*. *viridis* were evenly arranged in 9 cm Petri dishes on a double layer of filter paper. The Petri dishes were moistened with 5 ml of sterile deionized water or the required test solution. Seed germination was evaluated at a day/night temperature of 35/25°C for *A*. *retroflexus* and 30/20°C for *A*. *viridis* under a 12-h light/12-h dark regime, unless otherwise specified. The Petri dishes were sealed in zip-lock plastic bags to avoid loss of moisture. The number of seeds, replications, and the quantity of water or appropriate solute were the same for every experiment. The germination assessment was continued up to 28 days, and total cumulative germination was recorded, and the number of germinated seeds was converted into a germination percentage for each replication.

### Germination studies

The germination status of all populations was tested at the time of harvest, followed by the germination procedure described in the germination protocol section. The germination status was checked every 30 days after harvest until germination improved to more than 90%. During the study, seeds of both species were stored at room temperature (25°C) in separate paper bags, and seeds with a low level of dormancy were used in studies 1 and 2.

### Study 1: Effect of environmental conditions on germination and emergence of A. retroflexus and A. viridis

#### Temperature and light

In a factorial arrangement (species × population × temperature × light), the effects of five different alternating temperature regimes of 15/5, 20/10, 25/15, 30/20, and 35/25°C under different light treatments (12-h photoperiod and continuous darkness) on seed germination of *A*. *retroflexus* and *A*. *viridis* were studied. The seeds were incubated in one of the five growth chambers separately. The growth chambers were calibrated to provide the 12 h/12 h light/dark period. In continuous dark conditions, the Petri dishes were covered with two layers of aluminum foil to create a dark environment.

#### Osmotic and salt stress

A germination test was performed to evaluate the effect of osmotic potentials of 0.0, -0.1, -0.2, -0.4, -0.8, and -1.6 MPa on *A*. *retroflexus* and *A*. *viridis* populations. To obtain these osmotic potential concentrations, an appropriate amount of polyethylene glycol 8000 was dissolved into distilled water, following the method described by Michel and Radcliffe [28]. The effect of salt stress was determined using six concentrations (0, 50, 100, 150, 200, and 250 mM) of sodium chloride (NaCl). An appropriate amount of NaCl was dissolved into distilled water to prepare different NaCl concentrations [29].

#### Burial depth

The effect of seed burial depth on seedlings emergence of *A*. *retroflexus* and *A*. *viridis* populations was determined by placing seeds at soil depths of 0 (soil surface), 1, 2, 4, 6 and 8 cm. The experiment was conducted in a rainout shelter facility of the University of Queensland, Gatton. A grey vertosol soil (bulk density 1.32 g cm^-3^, pH 7.2, and organic matter 2.3%) was used. The soil was sieved using a 4 mm sieve and oven-dried at 100°C for 96 h to kill seeds in the soil [30]. Plastic pots (10 cm diameter × 10 cm height, 1 L) were filled with soil, and 50 seeds per pot were used. Pots were sub-irrigated during the experiment. The criteria for emergence was set as the appearance of two cotyledons.

### Study 2: Seedbank persistence of *A*. *retroflexus* and *A*. *viridis*

A field study was conducted at the Research Farm of the University of Queensland, Gatton, Australia, to evaluate the germinability and seedbank depletion of *A*. *retroflexus* and *A*. *viridis*. The experiment was a factorial arrangement of species, biotype, burial depth and burial durations arranged in a randomized complete block design. Fifty seeds of both populations of *A*. *retroflexus* and *A*. *viridis* were placed in separate permeable nylon bags (5 cm by 4 cm). Permeable bags were used to create conditions close to natural soil conditions (water and air diffusion and microorganisms). The bags were placed at 0 (soil surface), 2, and 10 cm of soil depths. Wooden sticks were used to tie the surface bags to stop the wind from moving them away. The bags were exhumed every three months (three replications), and the seeds were retrieved from the bags and cleaned in the laboratory before the germination tests. The seeds that lost their structural rigidity were labelled as deteriorated, decayed, or germinated. The viability of remaining seeds was evaluated using the germination test described in the germination protocol section. The viability of seed embryos of non-germinated seeds was determined by the crush test [31, 32]. This study was continued until 24 months after seed placement.

### Data analyses

All germination tests were laid out in a completely randomized design with three replications and were repeated once after completion of the first run. The normality (Shapiro-Wilk) and homogeneity (Breusch-Pagan) assumptions of the data were checked, and the original data was used. The significance of any treatment and their interaction with each other was evaluated using analysis of variance (ANOVA) (GENSTAT 16th edition; VSN International, Hemel Hempstead, United Kingdom) at a probability level of 0.05. In the first study, no significant differences were observed between the runs and treatments; therefore, data was pooled across the experimental runs (n = 6). In both studies, the data was also pooled across the populations as no significant differences were observed between populations (therefore, n = 12). After combination of insignificant variables, again the data was subjected to ANOVA and means were compared. The mean germination data of the temperature, light, burial depth and dormancy experiments were compared using the least significant difference (LSD) test at a probability level of 0.05. A three-parameter sigmoidal model was fitted to the germination data of salt stress and water potential experiments (Eq 1) using SigmaPlot 14 (Systat Software, San Jose, CA, United States).

$$F=\frac{X_{max}}{1+e\;\frac{-(X-X_{50})}{b}}$$ (1)

*F* is germination (%) or seed depletion (%) at a given osmotic potential, salt concentration or months after seed placement in the field; *X*_*max*_ is the maximum germination (%) or seed depletion (%); *X*_*50*_ is the osmotic potential, salt concentration or months after seed placement, which inhibited the maximum germination by 50% or depleted 50% of seeds; and *b* is the slope. A three-parameter logistic model was fitted to the seedbank germination data as follows:

$$F=\frac{X_{max}}{1+({\frac{T}{T_{50}})}^{b}}$$ (2)

*F* is germination percentage at a given months after seed placement (*T*); *Xmax* is the maximum germination; *T*_*50*_ is the months after seed placement at which the maximum viability (%) reduced by 50% and *b* is the slope. Parameter estimates of the salt, osmotic stress, seed viability, and seed depletion experiments were compared using the standard error of means.

## Results

### Germination studies

**Fig 2:**
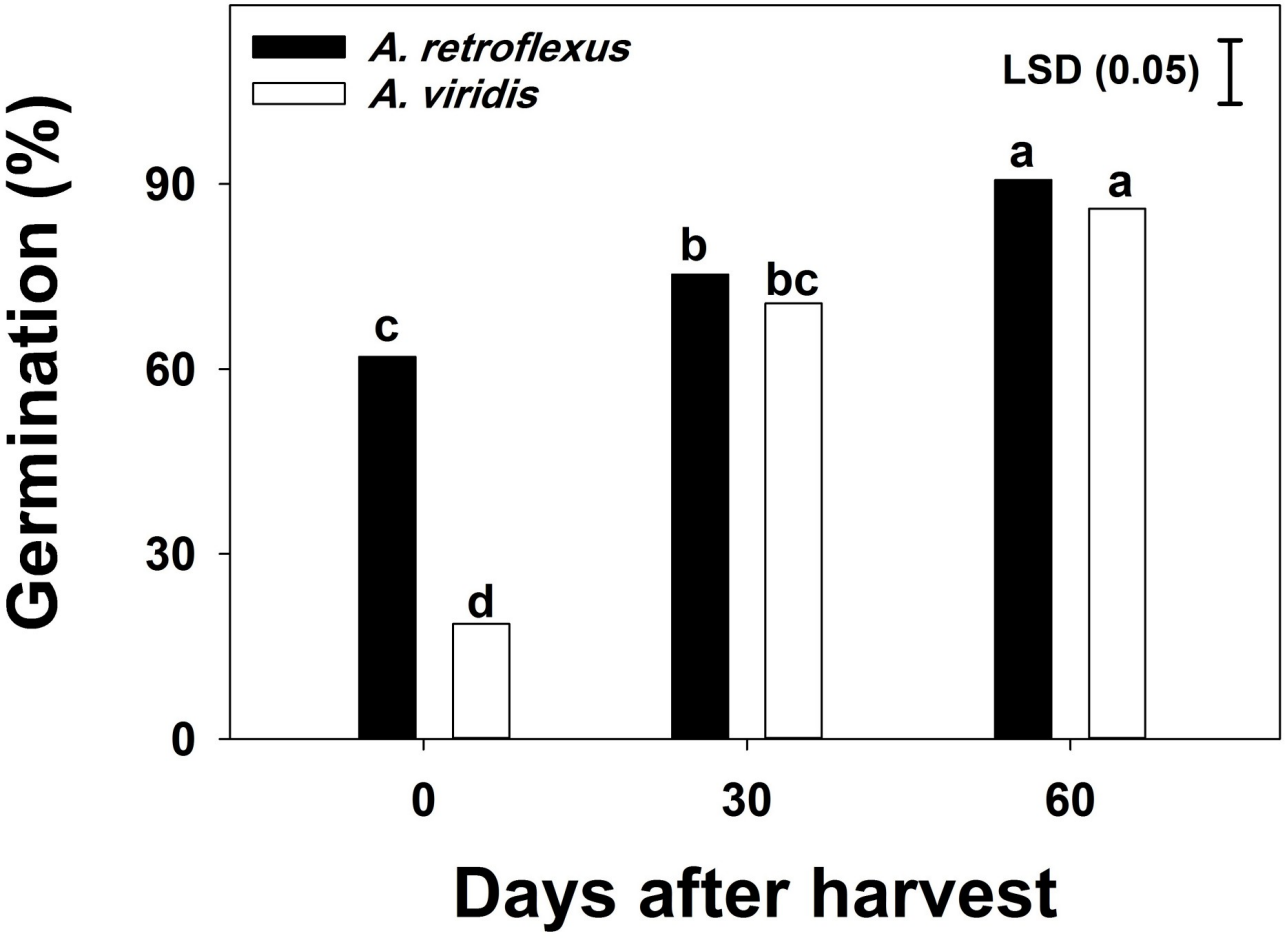
Seed germination of *Amaranthus retroflexus* and *Amaranthus viridis* after harvest. Each bar of the graph shows the mean value of data [pooled across populations (Gatton and Goondiwindi) and experimental repeats, n = 12]. Vertical bars depict the least significant difference (LSD) values at the 5% level of probability and the letters above bars show group differences between means.

No differences were observed between populations in either species. The effect of seed storage and weed species and their interactions was significant (*p<0*.*05*) on germination. Immediately after seed harvest, low germination was observed in *A*. *viridis* seeds (19%), while 62% of *A*. *retroflexus* seeds germinated (Fig 2). After two months of storage, no significant differences were observed between weed species; germination of *A*. *retroflexus* and *A*. *viridis* reached 93% and 86%, respectively.

### Study 1: Effect of environmental conditions on germination and emergence of *A*. *retroflexus* and *A*. *viridis*

#### Temperature and light

Germination was affected (*p* < 0.05) by the effect of weed species, alternating temperature, light treatment and their interactions (Table 1). Both species showed very low germination (< 1%) at 15/5°C. In the day/night temperature range of 25/15 °C to 35/25 °C, both weeds had more than 33% germination under different light treatments. In a 12-h photoperiod, the highest germination of *A*. *retroflexus* (93%) and *A*. *viridis* (86%) was observed at 35/25 and 30/25 °C day/night temperature, respectively. Although lower germination was observed in the dark treatment, it was not an inhibiting factor for the germination of either species.

**Table 1:** The effect of alternating day/night temperatures and light treatments on germination of *Amaranthus retroflexus* and *Amaranthus viridis* (study 1).

Species	Light treatments	Germination (%)				
		15/5°C	20/10°C	25/15°C	30/20°C	35/25°C
*A*. *retroflexus*	Light / dark	0.7	28.5	65.5	81.8	93.3
	Dark	0.3	19.8	47	64.5	80.5
*A*. *viridis*	Light / dark	0.7	8.3	51.7	85.7	73.0
	Dark	0.0	6.0	33.5	64.2	53.2
		LSD (0.05) = 6.36				

Data was pooled across populations (Gatton and Goondiwindi) and experimental runs (n = 12).

#### Osmotic and salt stress

**Fig 3:**
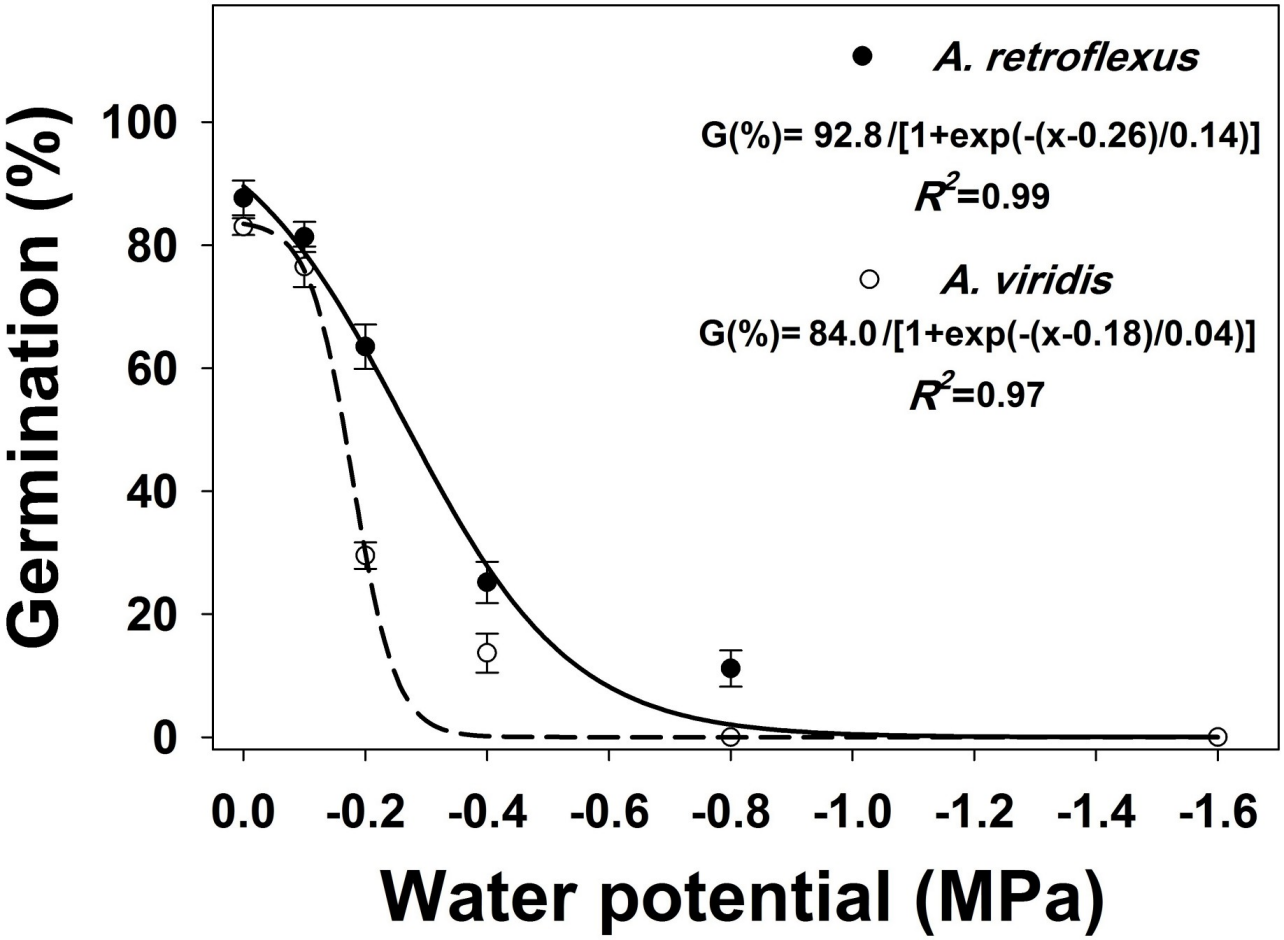
Effect of salt stress on germination of *Amaranthus retroflexus* and *Amaranthus viridis*. Each point of the graph shows the mean value of data [pooled across populations (Gatton and Goondiwindi) and experimental repeats, n = 12]. A three-sigmoidal model was fitted to data. Vertical bars show the standard error of means (study 1).

Osmotic potential, weed species, and their interactions significantly (*p* < 0.05) affected seed germination of both weed species. Germination declined with decreasing osmotic potential, and the greatest germination of both species was observed at 0 MPa (Fig 3). A three-parameter sigmoid model estimated that the germination of *A*. *retroflexus* and *A*. *viridis* was inhibited by 50% (*X*_*50*_ parameter) at osmotic potentials -0.26 and -0.18 MPa, respectively. At the osmotic potential of -0.8 MPa, *A*. *viridis* germination was completely inhibited, while 11% germination was observed for *A*. *retroflexus* at this osmotic potential.

**Fig 4:**
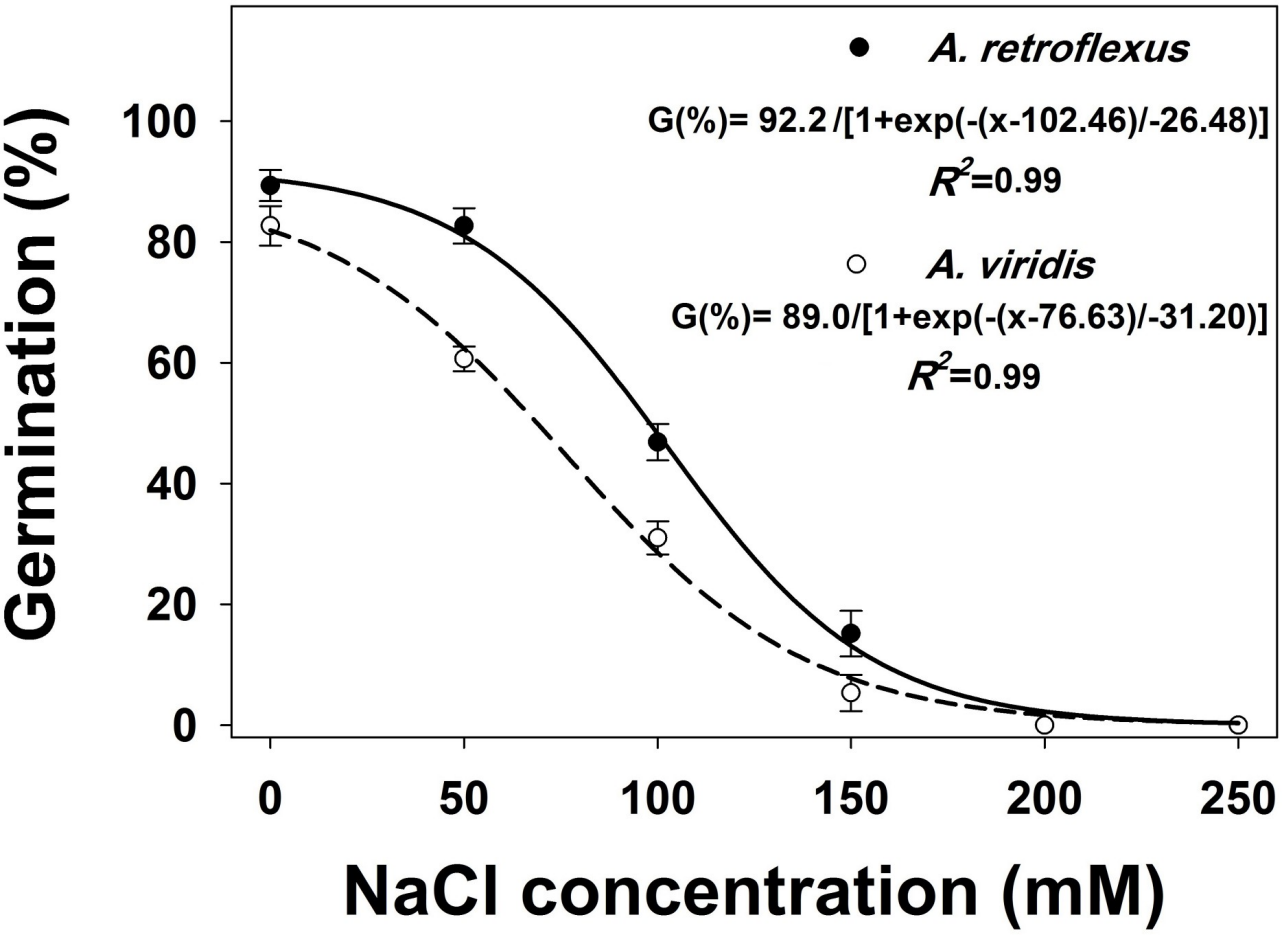
Effect of osmotic potential on germination of *Amaranthus retroflexus* and *Amaranthus viridis*. Each point of the graph shows the mean value of data [pooled across populations (Gatton and Goondiwindi) and experimental repeats, n = 12]. A three-sigmoidal model was fitted to data. Vertical bars are the standard error of means (study 1).

Germination was significantly (*p* < 0.05) influenced by salt stress, weed species and their interactions. Germination was highest in no stress conditions and germination declined with increasing NaCl concentrations (Fig 4). NaCl concentrations of 102 and 77 mM inhibited 50% germination of *A*. *retroflexus* and *A*. *viridis*, respectively. No germination was observed in both species at the NaCl concentration of 200 mM and above.

#### Burial depth

**Fig 5:**
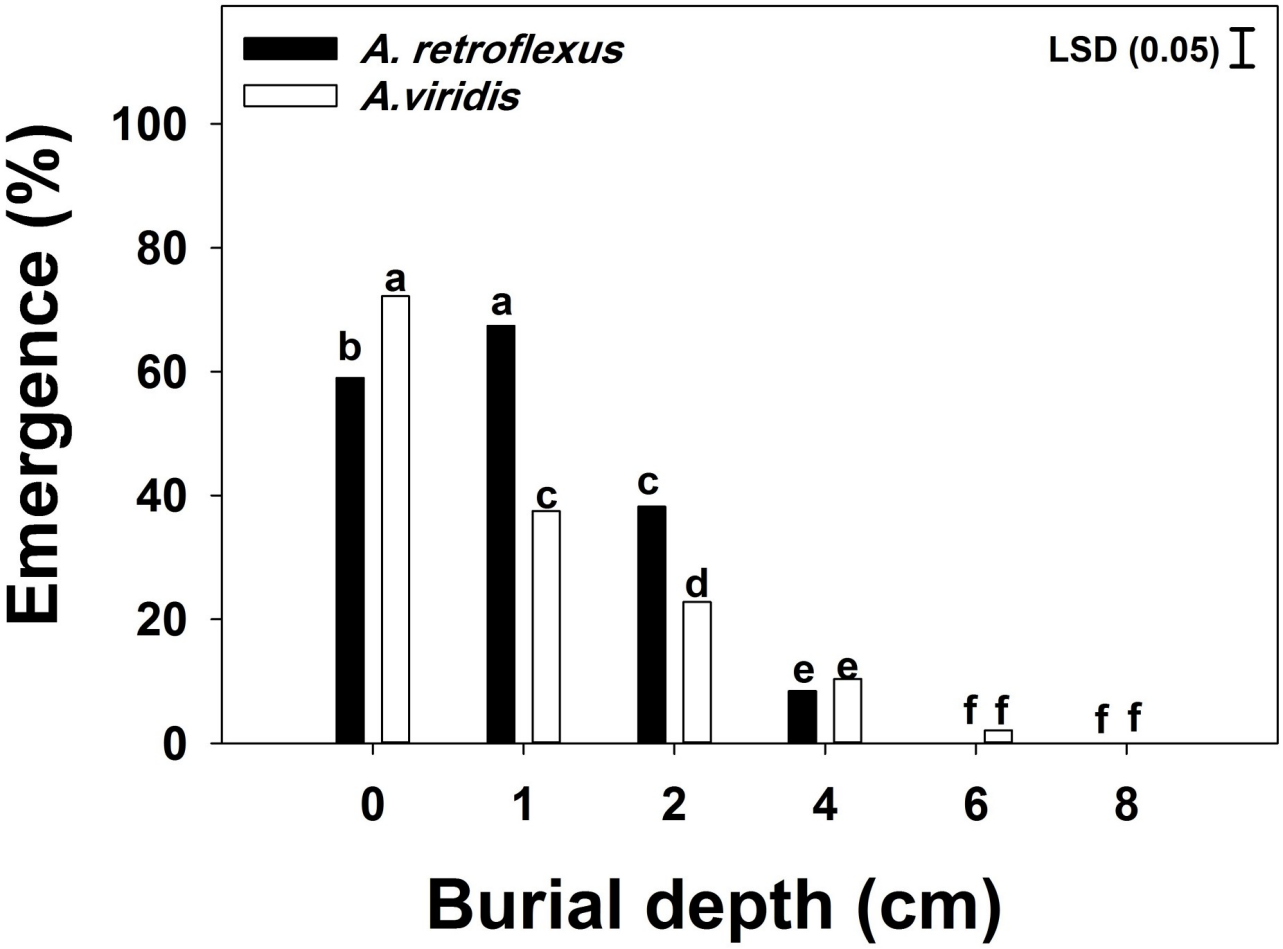
Effect of burial depth on seedling emergence *Amaranthus retroflexus* and *Amaranthus viridis*. Each bar of the graph shows the mean value of data [pooled across populations (Gatton and Goondiwindi) and experimental repeats, n = 12]. Vertical bars depict the least significant difference (LSD) values at the 5% level of probability and the letters above bars show group differences between means (study 1).

Seedling emergence was influenced (*p* < 0.05) by the seed burial depth, weed species, and their interaction. The highest emergence of *A*. *retroflexus* (67%) was observed from 1 cm burial depth, while the highest germination of *A*. *viridis* (72%) was observed on the soil surface (Fig 5). A sharp decline in emergence was observed with increasing burial depths, and the emergence of both species was less than 10% from 4 cm. The emergence of both species statistically was completely inhibited at 6 cm burial depth.

### Study 2: Seedbank persistence of *A*. *retroflexus* and *A*. *viridis*

**Fig 6:**
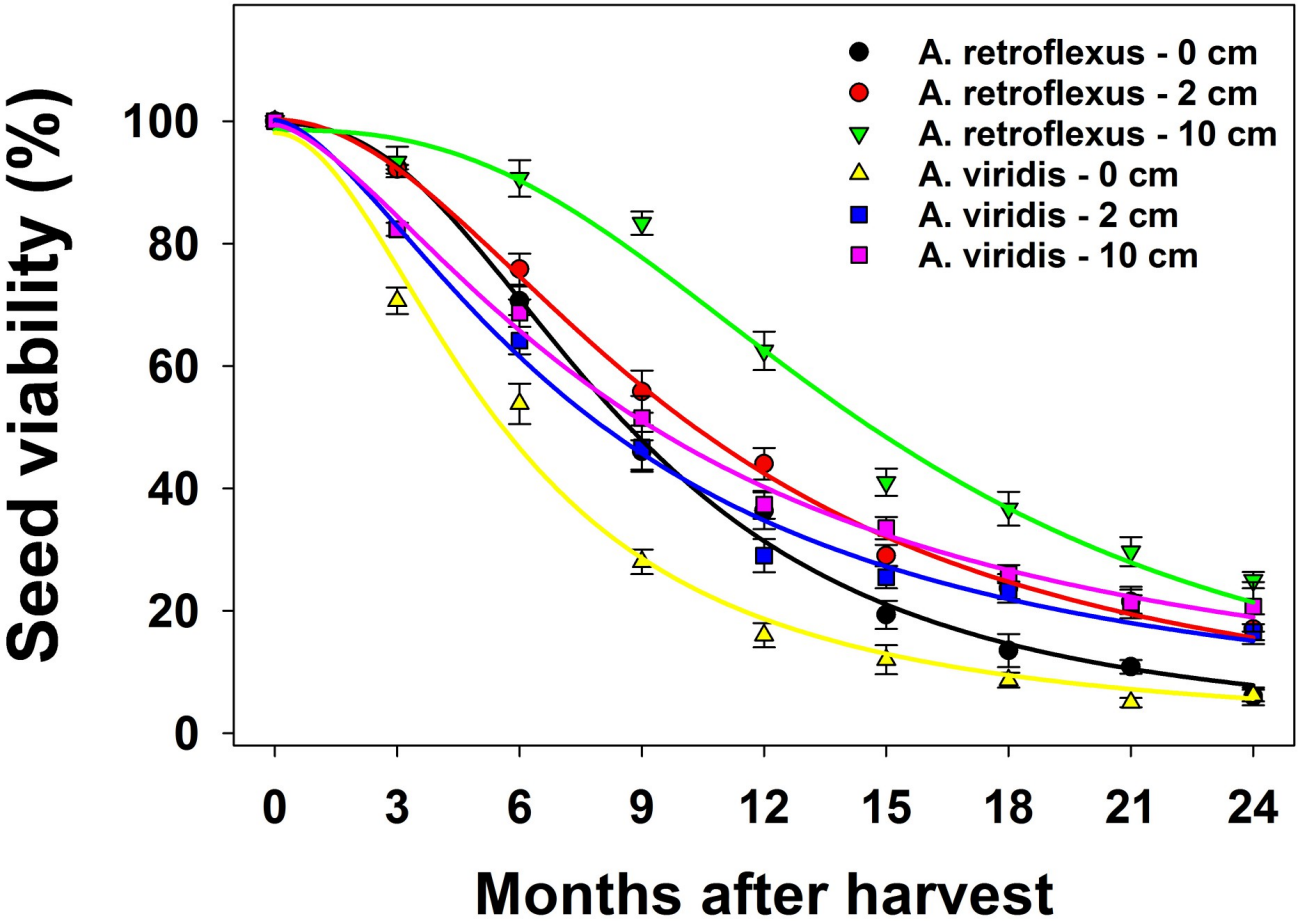
Effect of burial depth and burial duration on germination of *Amaranthus retroflexus* and *Amaranthus viridis*. Each point of the graph shows the mean value of data [pooled across populations (Gatton and Goondiwindi), n = 6]. A three-logistic model was fitted to data. Vertical bars depict the standard error of means. The estimated parameters are given in Table 1 (study 2).

The effects of weed species, burial depth, burial duration and their interactions on seed viability was significant (*p* < 0.05). In both species, regardless of the spatial position of seeds, seed viability was reduced over time and was lowest at 24-months after seed placement (Fig 6; Table 2). *A*. *retroflexus* seeds that were buried at 0, 5, and 10 cm lost 50% of their maximum viability (*T*_*50*_ parameter) at 9, 10, and 15-month after seed placement, respectively. Similarly, the maximum seed viability of *A*. *viridis* was reduced by 50% at 6, 8, and 9-mo after seed placement, respectively.

**Table 2:** A three-parameter logistic model fitted to viability (%) of *Amaranthus retroflexus* and *A*. *viridis* at different burial depths and burial duration (study 2).

Species and burial depth	Parameters			
	*X*_*max*_ (%)	*T*_*50*_ (month)	*b*	*R* ^*2*^
*Amaranthus retroflexus*				
0	99.6 (1.09)	8.72 (0.29)	2.43 (0.14)	0.99
5	98.6 (1.66)	10.28 (0.29)	1.99 (0.09)	0.99
10	98.6 (1.18)	14.79 (0.67)	2.65 (0.32)	0.98
*Amaranthus viridis*				
0	98.1 (1.99)	5.70 (0.44)	1.94 (0.19)	0.99
5	97.2 (2.96)	8.05 (0.49)	1.58 (0.12)	0.99
10	99.4 (1.01)	9.31 (0.39)	1.53 (0.08)	0.99

Values presented in the parentheses are standard error of means (±SE).

*G(%) = X*_*max*_ /(1*+* (*T/T*_*50*_)^*b*^), *G(%)* is germination percentage at given months after harvest *T*, *Gmax* is the maximum vability; *T*_*50*_ is the months after harvest, which the maximum vability (%) reduced by 50% and *b* is the slope.

**Fig 7:**
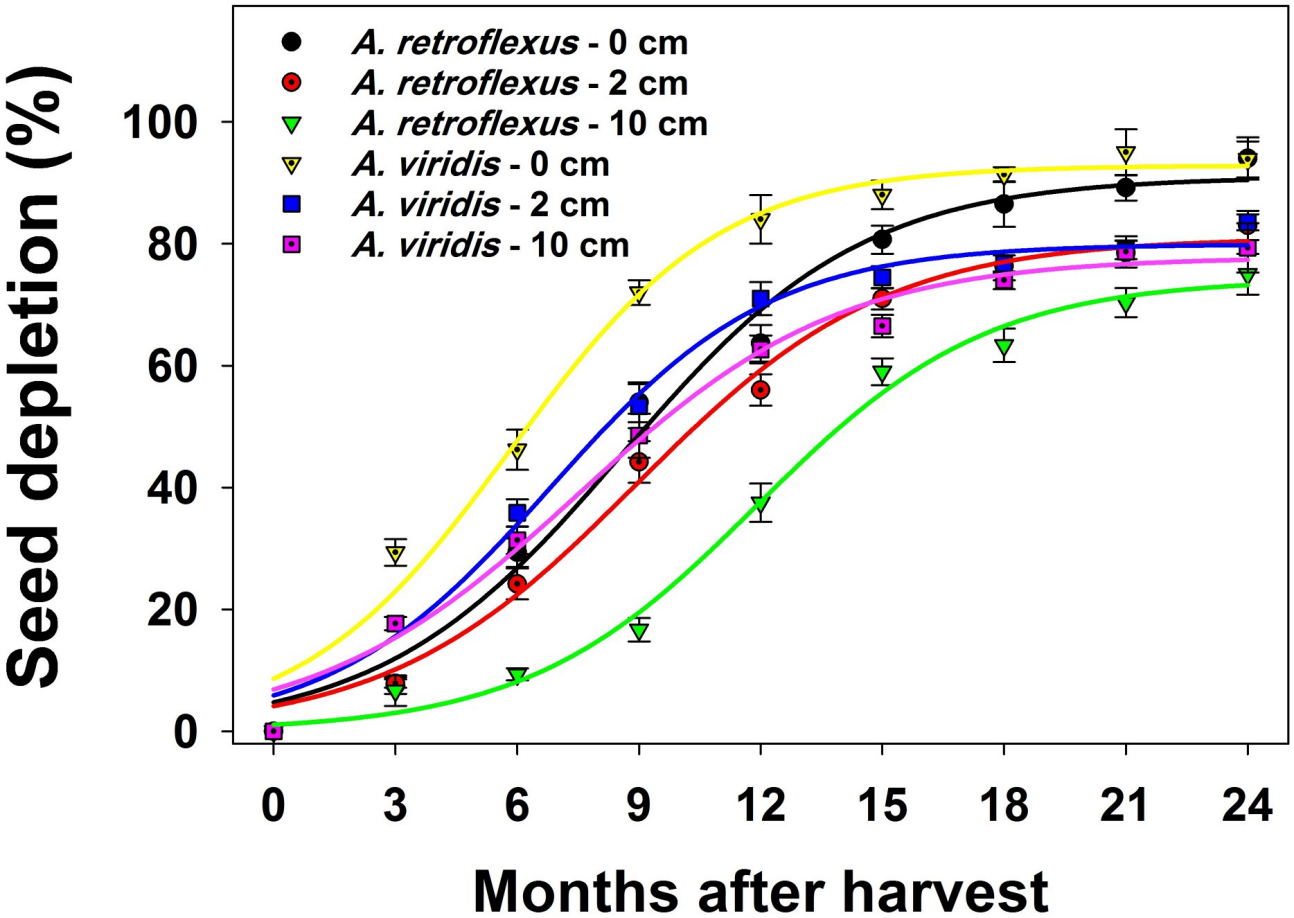
Effect of burial depth and burial duration on seed depletion of *Amaranthus retroflexus* and *Amaranthus viridis*. Each point of the graph shows the mean value of data [pooled across populations (Gatton and Goondiwindi), n = 6]. A three-sigmoid model was fitted to data. Vertical bars depict the standard error of means. The estimated parameters are given in Table 2 (study 2).

Weed species, seed burial depth, burial duration, and their interactions had a significant (*p* < 0.05) effect on the seedbank depletion. Irrespective of seed spatial position, seed depletion of both species was increased over time (Fig 7; Table 3). At 24-mo after seed placement, seed depletion of *A*. *retroflexus* ranged from 75% (10 cm depth) to 94% (soil surface). Similarly, seed depletion of *A*. *viridis* ranged from 79% to 94% at this time. Compared to seeds that were buried at 10 cm, the seed depletion of both species was higher on the soil surface. *A*. *retroflexus* seeds that were buried at 0, 5, and 10 cm showed a 50% depletion of seeds from the seedbank at 9, 9, and 12 months after seed placement, respectively. Similarly, 50% of *A*. *viridis* seeds were depleted from the seedbank at 6, 7, and 8 months after seed placement, respectively.

**Table 3:** A three-parameter sigmoid model fitted to seed depletion (%) of *Amaranthus retroflexus* and *A*. *viridis* at different burial depths and burial duration (study 2).

Species and burial depth	Parameters			
	*X*_*max*_ (%)	*X*_*50*_	*b*	*R* ^*2*^
*Amaranthus retroflexus*				
0	91.0 (3.09)	8.59 (0.47)	2.96 (0.40)	0.99
5	81.0 (2.18)	8.93 (0.37)	3.06 (0.31)	0.99
10	74.2 (2.51)	11.91 (0.40)	2.83 (0.32)	0.99
*Amaranthus viridis*				
0	92.8 (2.54)	5.86 (0.40)	2.58 (0.36)	0.99
5	79.9 (1.99)	6.82 (0.36)	2.69 (0.31)	0.99
10	77.8 (2.53)	7.50 (0.47)	3.21 (0.42)	0.99

Values presented in the parentheses are standard error of means (±SE).

*F = Xmax*/(1+exp(-(*X-X*_*50*_)/b)), F is seed depletion (%) at given months after seed harvest *X*, *Xmax* is the maximum seed depletion (%); *X*_*50*_ is months after seed harvest, which 50% of seed depletes and *b* is the slope.

## Discussion

The results showed that both *Amaranthus* species exhibited primary dormancy, which eased with an after-ripening period. Dormancy is responsible for the lack of germination in viable seeds in favorable conditions, which facilitates seed dispersal over time and location [23]. Cristaudo et al. [33] reported that after-ripening is a common phenomenon in *Amaranthus* species. Initial dormancy of these species may result in periodic germination over the growing season, which could lead to failures in predictions of the germination times and intensities [34, 35].

Temperature is an important factor that impacts seed germination by regulating activities of different enzymatic systems and hormone synthesis pathways that are responsible for the germination of seeds [23]. In this study, the effect of various alternating day/night temperatures on both weeds showed that *A*. *retroflexus* and *A*. *viridis* could germinate considerably between the temperature range of 25/15°C to 35/25°C. Low germination of both species in day/night temperature ranges of 15/5°C and 20/10°C could be a mechanism to ensure that the germination of these weeds only occurs in favorable conditions. Guo and Al-Khatib [21] reported that the germination of *A*. *retroflexus* was highest at a day/night temperature 35/30°C, and no seed germination was observed at 15/10°C. Similarly, the highest germination of *A*. *viridis* was observed in the temperature ranges of 30/20 to 35/25°C [15]. In cotton-growing regions of Australia (New South Wales and Queensland), except for the short winter season, the temperature ranges of 15 to 35°C is favorable for the germination of these species (Fig 1). It could be concluded that *A*. *retroflexus* and *A*. *viridis* could germinate in warm cotton farming regions of Australia and create problems for most summer season crops. The persistent seedbank, initial dormancy, and ability to germinate in a wide range of temperatures may result in periodic germination of this weed during the growing season [18–19]. Therefore, seeds of these species could theoretically germinate in several flushes and may escape from conventional weed management strategies.

Although light conditions significantly improved the germination of both species, the light was not an absolute requirement for the germination of *A*. *retroflexus* and *A*. *viridis*. Cristaudo et al. [33] reported that although the dark did not inhibit the germination of *A*. *viridis*, maximum germination occurred in the presence of light. In *Amaranthus* species, a cyclic change in the requirement of temperature and light has been reported over time, but the darkness could not inhibit the germination completely [34]. These findings suggest that *A*. *retroflexus* and *A*. *viridis* are capable of germinating even in dark conditions, allowing germination of these species to occur under a crop canopy or shallow soil depths.

A previous study in the Philippines also reported the lack of *A*. *viridis* germination at a water potential -0.8 MPa [15]. Hao et al. [5] showed that germination of the Chinese populations of *A*. *retroflexus* and *A*. *viridis* completely inhibited at -0.8 and -0.4 MPa, respectively. Although the germination of these species may be inhibited under high osmotic stress, the abundant summer rainfall in cotton-growing regions of Australia (New South Wales and Queensland) may explain the presence of these species. The lack of germination under high osmotic potential could be a defense mechanism for these species to survive under drought conditions [15].

Our study showed that germination of both species was completely inhibited at 200 mM NaCl. Similarly, no germination was observed in the Chinese populations of *A*. *retroflexus* and *A*. *viridis* at 200 mM NaCl [5]. Drought, high evaporation, and low rainfall conditions may result in the accumulation of salts in the soil profile. In Australia, 30% of the land is saline, and more than 60% of the agricultural area is potentially associated with saline water irrigation [36]. Our study suggests that germination of these species is not sensitive to salt stress, and these species could sustain their germination under moderate saline conditions.

In the current study, the adverse effect of the burial depth on the emergence of these species was observed. Information from studies conducted on other *Amaranthus* species indicates a similar trend of germination and emergence owing to the restricted carbohydrate supplies available in small-seeded broadleaf weeds [5, 15, 25]. Ghorbani et al. [25] studied the emergence trends of *A*. *retroflexus* and found that the optimum burial depth was between 0.5 and 3 cm, with no emergence from 5 cm. As *A*. *retroflexus* and *A*. *viridis* seedlings could not emerge from burial depths greater than 4 cm, deep tillage could be used as an option to halt their emergence.

Not all viable seeds germinate due to dormancy and unfavorable conditions. However, as long as seeds are viable in a seed bank, there is a risk of weed emergence and reproduction [23]. Korres et al. [16] reported that regardless of burial depth, only 4–5% of Palmer amaranth (*Amaranthus palmeri* S. Watson) and waterhemp [*Amaranthus tuberculatus* (Moq.) J. D. Sauer] seeds were viable after 3-yr. Although seed viability is largely related to plant species and environmental conditions, our results suggest that the inclusion of fallow conditions in crop rotation could be beneficial as up to 50% seed loss per year was observed.

Our results showed that the burial of seeds inhibited the emergence of both species, but it resulted in increased seed persistence. Steckel et al. [19] reported that tillage increased seed persistence of common waterhemp (*Amaranthus rudis* J. D. Sauer). The results of the current study showed that the emergence of *A*. *retroflexus* and *A*. *viridis* occurred close to the soil surface. In the no-tillage systems, seeds remain on the soil surface, and it has been reported that the emergence of weed species was higher in such systems in comparison to tillage systems [19]. It could be concluded that in no-tillage systems, farmers may face more infestation compared to tillage systems initially, but adopting no-tillage systems could be contributing to better weed seedbank management.

Avoiding seed bank replenishment and declining seed density is one of the most effective weed management strategies [23, 37]. The results of the current study showed that seed depletion (germinated or destroyed) of both species was higher on the soil surface compared to buried seeds. On the soil surface, seeds experience greater temperature and mositure fluctuations, seed predation, and microbial degradation [17]. In this study, the factors that influenced seed depletion were not studied; therefore, further studies are required to maximize the seed depletion of these species.

In the present study, the populations of the two *Amaranthus* species responded similarly to the tested environmental conditions. Genetic diversity and diverse maternal conditions during seed maturity could be responsible for the different responses of *Amaranthus* species populations to environmental conditions [35]. In the current study, the maternal effect was removed by re-growing plants in the same environment. It could be concluded that the ability to germinate in a wide spectrum of environmental conditions and persistence over time could be an innate characteristic of *Amaranthus* species. Hao et al. [5] claimed that the germination potential of seeds may not make any difference in the invasiveness of *Amaranthus* species, but germination performances of these species may enhance the worldwide distribution of these species.

The current study adds valuable information to our understanding of the germination biology of these species, which will be helpful in the development of suitable management practices and modeling the control strategies for the potential spread of *A*. *retroflexus* and *A*. *viridis* before these weeds become problematic across Australia. The study results showed that *A*. *retroflexus* and *A*. *viridis* germinate over a wide variety of environmental conditions. The results showed that germination of both species could be restricted under high osmotic and salt stress. A shallow tillage together can be an effective management practice to diminish the soil seedbank in arable lands, but the seed burial depth increases the seedbank persistence. The findings of this study will help farmers and agronomists working on cotton and summer crops to build sustainable integrated weed management practices for effective management and control of *A*. *retroflexus* and *A*. *viridis*.
